# Direct and Indirect Costs of Asthma Management in Greece: An Expert Panel Approach

**DOI:** 10.3389/fpubh.2017.00067

**Published:** 2017-04-05

**Authors:** Kyriakos Souliotis, Hara Kousoulakou, Georgios Hillas, Petros Bakakos, Michalis Toumbis, Stelios Loukides, Theodoros Vassilakopoulos

**Affiliations:** ^1^Department of Social and Educational Policy, University of Peloponnese, Corinth, Greece; ^2^Department of Pulmonary and Critical Care Medicine, Medical School, Evangelismos Hospital, National and Kapodistrian University of Athens, Athens, Greece; ^3^1st Respiratory Medicine Department, “Sotiria” Hospital for Thoracic Diseases, University of Athens Medical School, Athens, Greece; ^4^6th Pulmonary Department, “Sotiria” Hospital for Thoracic Diseases, Athens, Greece; ^5^2nd Respiratory Medicine Department, “Attikon” Hospital, University of Athens Medical School, Athens, Greece

**Keywords:** asthma, burden, direct cost, indirect cost, Greece

## Abstract

**Objective:**

Asthma is a major cause of morbidity and mortality and is associated with significant economic burden worldwide. The objectives of this study were to map current resource use associated with the disease management and to estimate the annual direct and indirect costs per adult patient with asthma.

**Methods:**

A Delphi panel with seven leading pulmonologists was conducted. A semistructured questionnaire was developed to elicit data on resource use and treatment patterns. Unit costs from official, published sources were subsequently assigned to resource use to estimate direct medical costs. Indirect costs were estimated as number of work loss days. Cost base year was 2015, and the perspective adopted was that of the National Organization of Health Care Services Provision, as well as the societal.

**Results:**

Patients with asthma are mainly managed by pulmonologists (71.4%) and secondarily by general practitioners and internists (28.6%). The annual cost of managing exacerbations was estimated at €273.1, while maintenance costs were estimated at €1,100.2 per year. Total costs of managing asthma per patient per year were estimated at €2,281.8, 64.4% of which represented direct medical costs. Of the direct costs, pharmaceutical treatment was the key driver, accounting for 63.9 and 41.2% of direct and total costs, respectively. Direct non-medical costs (patient travel and waiting time) were estimated at €152.3. Indirect costs accounted for 28.9% of total costs.

**Conclusion:**

Asthma is a chronic condition, the management of which constrains the already limited Greek health care resources. The increasing prevalence of the disease raises concerns as it could translate per patient costs into a significant burden for the Greek health care system. Thus, the prevention, self-management, and improved quality of care for asthma should find a place in the health policy agenda in Greece.

## Introduction

Asthma is an obstructive airway disorder and one of the most common chronic diseases in the world (1, 2). It has been estimated to affect 300 million individuals worldwide (3), and about 30 million children and adults younger than 45 years have asthma in Europe (4). In USA, asthma prevalence increased from 7.3% in 2001 to 8.4% in 2010 (5), while in Greece, it has been estimated at 8.6% of the general population (6). Asthma is a global public health problem, and the World Health Organization reports that it affects all countries regardless of level of development (7).

Although asthma is associated with low mortality rates (8), it is a major cause of morbidity and is associated with significant economic burden. The number of years lived with disability worldwide was estimated at 15.9 million in 2015 (9). The economic cost of asthma is considerable in terms of both direct medical (hospitalization and pharmaceutical care) and indirect costs (productivity losses) (1). A recent review showed that annual per patient costs in the published literature varied from less than US$150 in Abu Dhabi to more than US$3,000 in USA (10). Indirect costs per year have been estimated at US$426 and US$1,154 per patient in USA and Europe, respectively (11, 12).

Despite the size of asthma prevalence in Greece, there is scarce evidence quantifying the economic burden of the disease. In the literature, only one study, dating back to 1997, was identified, which aimed at estimating both direct and indirect costs associated with the management of the disease (13). The study reported total direct medical costs of 15.5 billion drachmas (approximately €44 million in 1997 prices) and indirect costs of 14.2 billion drachmas (approximately €41.7 million in 1997 prices). The study also reported 800,000 asthma-attributed work days lost (13). Since that publication, direct and indirect costs in the country have not been adequately explored, despite changing disease management strategies. The objective of this study was to provide recent estimates of the direct and indirect costs of asthma management in adult patients in Greece, by experts mapping current resource use associated with disease management.

## Materials and Methods

A Delphi panel with seven leading pulmonologists, members of the Asthma Working Group of the Hellenic Thoracic Society, was conducted. The members of the panel were selected on the basis of experience (≥15 years) and number of patients with asthma they currently manage (≥50 per month) and represented all settings of health care provision in Greece (public and private hospitals, University hospitals, and private physician office). All experts were selected from the Attica Health Care District, currently covering almost half the Greek population, and were asked to provide their opinion on real-world data, i.e., to describe how asthma is currently being managed in the country.

To map local treatment patterns and resource use for the management of asthma, a semistructured questionnaire was developed with input from the international literature, which was validated by clinical experts. The questionnaire consisted of questions on direct medical costs (pharmaceutical, medical, hospital care, as well as lab and functional/imaging tests), direct non-medical costs (patient travel and waiting time to receive medical care), and indirect costs (non-paid caregivers’ time and work loss days due to sick leave prescribed by the physician). The use of additional resources (oxygen therapy and nebulizers at home) was also recorded. All resource use components were collected for the maintenance phase and exacerbations.

The questionnaire completion method was the Delphi technique, which provides mean values for each parameter by achieving convergence of opinion through multiple rounds of completion (14). The process was conducted between October 2015 and January 2016, and consensus was reached during the second round of completion.

Total costs were subsequently calculated by assigning unit costs on resource use data collected *via* the expert panel. Direct medical costs were estimated from the perspective of the National Organization of Health Care Services Provision (EOPYY), which currently covers more than 90% of the Greek population (15). The cost of pharmaceutical treatment was estimated based on reimbursement prices (16); hospitalization costs were based on the diagnosis-related groups (DRGs) (17); intensive care unit (ICU) costs were based on the Decree published by the Ministry of Health (18); lab and imaging test costs were estimated with prices reimbursed by EOPYY (19); and the cost of additional resources was calculated based on the Unified Regulation for Health Care Benefits (EKPY) (20). The cost base year was 2015. Indirect costs were estimated based on Organization for Economic Cooperation and Development data for Greece’s per capita gross domestic product (GDP), projected to 2015 based on historical average annual growth rate (2008–2014) (21). All cost estimates refer to the average patient, i.e., have been weighted with the probability of use of each resource item. In addition, the average waiting time has been weighted with the respective percentages of patients visiting physicians’ private offices and hospital outpatient clinics.

## Results

Patients with asthma are mainly managed by pulmonologists (71.4%) and secondarily by general practitioners (GPs) and internists (jointly representing 28.6%). During the maintenance phase, the health care settings that patients most frequently visit are hospital outpatient clinics (49.1%) and physician’s private offices (45.7%), while 5.2% receive treatment at home. During exacerbations, visits at home are slightly increased (8.8%), while 29% of the patients also visit the hospital emergency rooms (ERs) (Table 1). Laboratory tests during both exacerbations and maintenance phase include complete blood count, biochemical tests, and urine analyses. Imaging tests include spirometry, electrocardiogram, chest X-ray, and computerized tomography. The frequency and percentage of patients undergoing each test per exacerbation and during the maintenance phase, as well as the unit costs per test, are presented in Table 1.

**Table 1:** **Resource use and unit costs**.

	Exacerbations		Maintenance phase		Unit cost (€)
	% of patients	Frequency per exacerbation	% of patients	Frequency per year	
A&E^b^	29.0	1.1	–	–	–
**Physician visits**					
Visit to hospital outpatient clinics^b^	45.3	1.2	49.1	14.3	–
Visit to physician’s private office	45.9	1.4	45.7		10
Physician’s visit at home^b^	8.8	1.2	5.5		–
**Laboratory tests**					
CBC	68.0	1.2	21.0	1.0	2.9
Biochemical tests^a^	42.0	1.2	15.0	0.8	21.7
Allergy blood testing (IgE + RAST)	–	–	70.0	1.0	23.8
Urine analysis	2.0	0.1	–	–	1.8
**Functional/imaging tests**					
Spirometry	64.0	1.2	94.0	1.3	44.0
Electrocardiogram	20.0	0.7	10.0	0.9	4.1
Chest CT	12.0	0.7	12.0	0.6	45.0
Chest X-ray	37.0	1.2	23.0	0.7	4.1

*CBC, complete blood count; IgE, immunoglobulin E; RAST, radioallergosorbent test; CT, computerized tomography*.

*^a^Including urea, creatinine, SGOT-SGPT-K-Na-Ca, and LDH*.

*^b^Not reimbursed by EOPYY*.

The breakdown of the total direct medical costs into its components is illustrated in Table 2. During the maintenance phase, the largest cost component is pharmaceutical treatment, which accounts for more than 80% of total maintenance cost. The Delphi panel results indicate that the majority of patients (81%) receive a combination of inhaled corticosteroids and long-acting beta agonists, while 9% receive ICS monotherapy. Thirty-six percent of all patients receive montelukast on top and irrespective of other pharmaceutical treatment. Contrary to the maintenance phase, during the exacerbation phase, pharmaceutical treatment accounts for a considerably smaller proportion (i.e., eightfold less) of total direct medical costs. The key cost driver is hospital and ICU admissions, which jointly account for more than half of the total exacerbation costs (Table 2).

**Table 2:** **Annual per patient costs during exacerbations and maintenance phase (€)**.

	Annual costs associated with the management of exacerbations^a^ (% of total)	Annual costs of the maintenance phase (% of total)
Pharmaceutical treatment	22.9 (8.4)	916.9 (83.3)
Medical treatment	10.3 (3.8)	65.1 (5.9)
Hospitalization	84.2 (30.8)	33.2 (3.0)
ICU	71.0 (26.0)	–
Lab tests	21.3 (7.8)	27.0 (2.5)
Functional/imaging tests	63.4 (23.2)	58.0 (5.3)
Total	273.1 (100.0)	1,100.2 (100.0)

*ICU, intensive care unit*.

*^a^Based on the mean number of exacerbations per year (1.6)*.

*Numbers have been rounded to the nearest euro, thus percentages might not sum up to 100%*.

The Delphi panel reported that only 10% of patients require hospitalization during the exacerbation phase and 40% of those hospitalized fall under the DRG code A29M (Bronchitis and asthma with comorbidities and/or complications), with an average length of stay (LOS) of 4 days and a cost of €792. The remaining 60% of patients is hospitalized under DRG code A29X (Bronchitis and asthma without comorbidities and/or complications), with an average LOS of 2 days and mean cost of €361. Of the patients hospitalized, 3.8% requires hospitalization in an ICU, with a LOS of 5.8 days and an additional cost of €1,167 per patient. Hence, the average cost per exacerbation—weighted with the probability of requiring hospitalization in an ICU—is estimated at €44.4, while the mean number of asthma exacerbations per patient is reported to be 1.6 per year. The cost of medical treatment appears to be very low as physician visits are reimbursed by EOPYY at €10 (22), although at private practice, out-of-pocket payments per visit may vary. Moreover, the management of asthma does not require significant use of additional resources. In particular, only 4.2 and 16.6% of asthma patients require nebulizer and oxygen therapy at home, respectively, with the average cost of these resources estimated at €96.6 per year.

The average patient time for medical follow-up and prescription-related visits to physicians, including travel and waiting time, ranged from 0.9 to 2 h for the private office and the hospital outpatient clinics, respectively (Table 3). Given that patients visit their physician approximately 1.2 times per month during the maintenance phase, the total number of medical follow-up visits was estimated at 14.3 per year, and thus, the mean patient time was estimated to range between 12.9 and 29 h per year. The mean annual cost of patient time (based on the mean daily wage rate and weighted for the percentage of patients per health care setting) was €152.3. The average number of work loss days per year was estimated at 10.5 (Table 4). In addition, approximately 2.7% of patients receive assistance from relatives and/or friends for their daily activities. This assistance results in caregivers’ time of approximately 2.1 h per day, 3.6 days per week with 75% of the caregivers being economically active.

**Table 3:** **Patient time costs per year**.

	Patient time (number of hours per visit)	% of patients
Private physician	0.9	51.8
Hospital outpatient clinic	2.0	48.2
Number of visits per year	14.3	
Daily (hourly) wage rate (€)	57.4 (7.2)	
Patient time cost (€)	152.3	

**Table 4:** **Indirect costs: cost of work loss days and cost of caregivers’ time per year**.

Daily (hourly) wage rate (€)	57.4 (7.2)
Work loss days due to asthma per year^a^	10.5
**Cost of work loss days (€)**	**602.6**
Percentage of patients requiring assistance from family/friends	2.7
Mean number of days per week	2.1
Mean number of hours per day	3.6
Percentage of caregivers who are economically active	75%
**Caregiver cost (€)**	**57.1**

*^a^Number of days of sick leave prescribed by the physicians*.

Table 5 illustrates the estimated sum and breakdown of the aforementioned direct medical, direct non-medical, and indirect costs. Direct non-medical and indirect costs account for 37% of total asthma-related costs.

**Table 5:** **Total per patient costs (€)**.

	Annual cost	% of total cost
**Direct medical cost^a^**	**1,469.8**	**64.4**
Pharmaceutical treatment	939.8	41.2
Medical treatment	75.4	3.3
Hospitalization and ICU	188.3	8.3
Lab and functional/imaging tests	169.7	7.4
Costs of additional resources	96.6	4.2
**Direct non-medical cost**	**152.3**	**6.7**
Cost of patients’ time	152.3	6.7
**Productivity losses**	**659.7**	**28.9**
Cost of work loss days	602.6	26.4
Cost of non-paid caregivers’ time	57.1	2.5
**Total cost**	**2,281.8**	**100**

*ICU, intensive care unit*.

*^a^Direct medical costs reflect both maintenance phase and exacerbation-related costs*.

*Numbers have been rounded to the nearest euro*.

## Discussion

This study explored the direct and indirect costs of asthma in Greece. Annual per patient costs were estimated at €2,282, with direct (medical and non-medical) and indirect costs accounting for 71 and 29% of total costs, respectively. The key driver of direct costs was pharmaceutical treatment (63.9%), whereas hospitalization (including admission to the ICU) was responsible for 8.3% of direct costs.

These findings are consistent with the international literature. Study findings suggest that medication costs are the largest cost component of direct costs in North America and Europe, ranging from 51% in USA to 68% in Canada and in Europe from 45% in Spain to 84% in Germany (11, 12, 23). On the other hand, there is evidence supporting a downward trend in rates and costs associated with hospitalization (23–25). This is attributed to the effective medication usage during the maintenance phase, resulting in fewer exacerbations and thus hospitalizations (26).

This trend along with the introduction of usage of LABA/ICS combinations during the maintenance phase could—at least partly—explain the discrepancies between our findings and the previous study conducted by Matsaganis et al. within the Greek health care setting (13). The latter had shown much lower than the current estimate costs for pharmaceutical care (35% of direct costs) and a much higher share of hospitalization costs (28%). However, that study is quite old, and treatment patterns, as well as major cost centers in the management of asthma, have significantly changed since then, not only in Greece but also globally. Over the 20-year gap between the two studies, many new medications have received marketing authorization for the management of asthma (mainly LABA/ICS), changing the treatment landscape and leading both to higher costs and improved effectiveness, which reduce the need for hospitalization. Recent findings suggest that pharmaceutical costs have been increasing in USA and Canada (10). In particular, in USA, medication costs have increased by 49% between 2000 and 2009 (24), while from 2001 to 2009, ER visits and hospitalization rates per 100 asthma patients remained stable (5).

Indirect costs estimated in our study account for almost one-third of total costs, and although they constitute a significant cost component, they are still lower than the respective estimate in the Matsaganis et al.’s study (48%) (13). This could be attributed mainly to the fact that in the latter study, work loss days were evaluated at the cost of labor, which was much higher at that time. Since Greece entered the era of economic crisis in 2008, the cost of labor and per capita GDP have been following a decreasing trend, leading to lower estimates of productivity losses.

Another interesting finding of this study is that contrary to what would be expected, the costs of exacerbations appear to be lower than the maintenance phase. This could be explained by the method of data collection and the structure of the health care system in Greece. Delphi panel data largely reflect the perceptions of physicians who typically monitor patients during the maintenance phase, whereas in the exacerbation phase, a considerable number of patients (almost 30%) seek care in the ERs of hospitals. In addition, there is evidence in the literature that asthma control before an exacerbation plays a role in the resource use and cost during exacerbations, with higher exacerbation costs in uncontrolled asthma patients (27). Thus, patients currently being managed by pulmonologists could be better controlled, thus reporting lower resource use during exacerbations.

Our study has certain limitations. First, all experts participating in the Delphi panel came from the Attica Health Care District; thus, it could be argued that results are not representative and could not be extrapolated to the national level, since health services in Greece are unequally distributed and patient pathways in rural areas may differ from those in Attica. However, Attica is the largest Health Care District in the country, covering almost half of the Greek population. Moreover, experts were selected as key opinion leaders, having the ability to describe the clinical practice in the country. They were asked throughout the questionnaire completion process to provide input regarding real-world knowledge, i.e., their opinion on how asthma is managed across the country and not only in their setting and district.

In addition, there are certain aspects of asthma-related costs that have not been explored in this study, such as social care, pulmonary rehabilitation, presenteeism, productivity loss due to early retirement, and shorter life expectancy. Although social care and pulmonary rehabilitation are very limited within the Greek health care setting and it would be expected to have a negligible impact on costs, the other cost components ought to be explored in future studies.

Our study is also based on expert opinion rather than patient level data; however, the Delphi technique is a widely used and accepted method for achieving convergence and is well recognized as a qualitative technique for data elicitation on resource use (14). In addition, the Delphi panel only included pulmonologists, although GPs also have an important role in the disease management, and did not include patients who could have provided a better estimate of non-medical direct (e.g., travel costs) and indirect costs (e.g., productivity loss) of asthma management. Therefore, the results of this study could be considered as a first step toward providing updated estimates of asthma management costs and should be supplemented by additional studies, exploring costs through patient surveys or case vignettes (28).

Overall, this study aimed at covering a literature gap of 20 years by capturing resource use and treatment patterns and updating estimates of direct and indirect cost associated with the management of asthma, through a validated process. Per patient asthma costs are high, and the increasing prevalence of the disease translates per patient costs into a significant burden for the Greek health care system. Therefore, asthma should be recognized as a priority disorder in government strategies and planning, and results of this study can feed into the discussions around cost rationalization policies in the field of health care.

## Conclusion

Asthma is a chronic condition, the management of which constrains the already limited Greek health care resources. The increasing prevalence of the disease raises concerns as it could translate per patient costs into a significant burden for the Greek health care system. Thus, the prevention, self-management, and improved quality of care for asthma should find a place in the health policy agenda in Greece.

## Ethics Statement

The study was approved (including methodology and ethics approval) by the Research Committee of the University of Peloponnese.

## Author Contributions

KS and HK designed the study, conducted the primary research, performed the analysis of data, and drafted the manuscript. GH, PB, MT, SL, and TV contributed to the study design, questionnaire development, conduct of the expert panel, and data analysis. All authors read and approved the final manuscript.

## Conflict of Interest Statement

The authors declare that the research was conducted in the absence of any commercial or financial relationships that could be construed as a potential conflict of interest.
